# A review of rice male sterility types and their sterility mechanisms

**DOI:** 10.1016/j.heliyon.2023.e18204

**Published:** 2023-07-13

**Authors:** Yusheng Xu, Dong Yu, Jin Chen, Meijuan Duan

**Affiliations:** a College of Agronomy, Hunan Agricultural University, Changsha 410128, China; b Hunan Hybrid Rice Research Center, Changsha 410125, China

**Keywords:** Rice, Heterosis, Male sterility, Mechanism, Multiplication system

## Abstract

Male sterility plays an important role in the utilization of heterosis in rice. The establishment of male sterile lines in rice is one of the key technologies in hybrid rice production systems. The currently widely used male sterile line breeding systems mainly include: three-line hybrid rice based on cytoplasmic male sterility, two-line hybrid rice based on environmental sensitive gene male sterility, and third-generation hybrid rice based on nuclear gene male sterility Seed production system. This study reviewed the types and mechanisms of male sterility in rice, and looked forward to the development direction of hybrid rice.

## 1 Introduction

Grain is an overall and fundamental strategic issue related to the social and economic stability of a country, and it is also an important cornerstone for ensuring national security. Rice (*Oryza sativa* L.), as a staple food for nearly half of the world's population, is one of the most important food crops in the world [1]. With the increase in the world population, the demand for rice is increasing, but at present, the rice planting area is gradually decreasing, and increasing rice yield per unit area is the key to solving the food problem [2]. Therefore, under the social background of increasing population year by year and declining arable land scale, increasing grain yield per unit area is the most direct way to solve the contradiction between increasing grain demand and decreasing cultivated land resources, and it is also the fundamental means to ensure China's food security. However, using rice male sterile materials to produce hybrid rice with heterosis is an effective way to increase rice yield. Therefore, the related research on male sterility in rice is of great significance to the cultivation of new hybrid rice varieties.

Male sterility refers to the abnormal development of stamens in higher plants in the process of sexual reproduction, resulting in pollen defects and unable to pollinate normally, while the pistil develops normally and can accept pollen to complete fertilization and fruiting. The discovery of rice male sterile materials solved the problems of self-pollination and artificial castration, and greatly promoted the utilization of rice heterosis., but the male sterile materials in rice could not reproduce through self-breeding. Therefore, solving the problem of reproduction of male sterile lines is an important link in the utilization of rice heterosis.

**Fig. 1:**
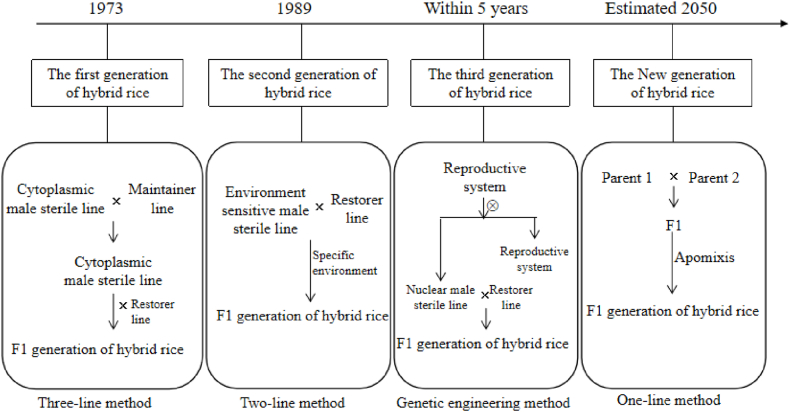
The development process of hybrid rice technology.

Rice is a typical monoecious and self-pollinated crop. Male sterile line is the core genetic tool to make use of heterosis. At present, the more mature male sterile line reproduction systems are mainly three-line hybrid rice based on cytoplasmic male sterility, two-line hybrid rice based on environmentally sensitive genic male sterility, and the seed production system of the third generation hybrid rice based on common recessive genic male sterility [3]. Actively explore the latest generation of hybrid rice breeding technology (single-line method) using apomictic technology to fix heterosis [4]. ((Fig. 1).

## 2 The different classification of male sterility in rice

Rice male sterility can be divided into classic abortion type, round abortion type, and dye abortion type according to the morphological characteristics of abortive microspores [[5], [6], [7], [8]]. The abortion stage of typical abortive pollen was earlier, the pollen stopped developing after developing to uninucleate pollen, there was no starch accumulation in the pollen, and can not be stained by I2-KI. Round abortive pollen abortion mainly occurred in the binucleate stage, the pollen grains were round, there was no or a small amount of starch accumulation, and I2-KI staining was not stained or light-colored. The pollen abortion of the dye-aborted male sterile line occurred at the trinucleate stage, there was a large amount of starch accumulation, and I2-KI staining was blue-black. According to whether fertility is regulated by the sporophytic gene or the gametophytic gene, rice male sterility can be divided into the sporophytic sterility and the gametophytic sterility. Sporophytic sterility is controlled by the genotype of the parent plant (sporophyte) without the pollen (gametophyte) genotype. Sporophytic sterility mostly occurs in the microsporogenesis stage of sporophytic stage. However, gametophytic sterility is controlled by gametophyte genotypes and has no relation to sporophytic genotypes. This pollen abortion occurs after meiosis of the pollen mother cell, the gametophytic stage. According to the source of sterile genes, rice male sterility can be divided into cytoplasmic male sterility (Cytoplasmic male sterility,CMS) and nuclear male sterility (Genic male sterility,GMS). However, according to whether its sterility is affected by the external environment, nuclear male sterility can be divided into environment-sensitive nuclear male sterility (Environment-sensitive genic male sterility, EGMS) and common nuclear male sterility (Spontaneous genic male sterility, SGMS).

## 3 Cytoplasmic male sterility

**Fig. 2:**
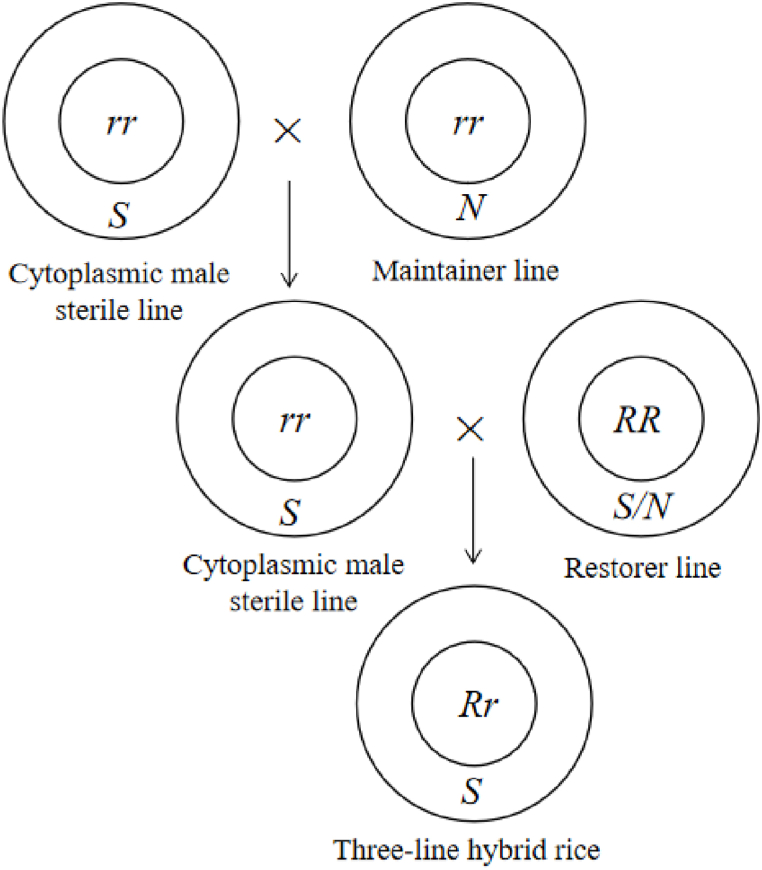
Schematic diagram of seed production system of three-line hybrid rice. s represents cytoplasmic sterility gene, N represents cytoplasmic fertility gene, r represents nuclear sterility gene, and R represents nuclear fertility restoration gene.

CMS is caused by uncoordinated expression of the mitochondrial genome and nuclear genome, interfering with normal plant metabolism, resulting in abnormal physiological reactions such as cytotoxicity, a disorder of energy metabolism, abnormal programmed cell death, and reverse regulation, resulting in pollen abortion [[9], [10], [11]]. In the production and application of hybrids, CMS was applied to the first-generation hybrid rice (three-line hybrid rice), in which the cytoplasmic gene of male sterile line contained sterile gene and the nuclear gene did not contain fertility-restoring gene, which showed male sterility. The maintainer plant cytoplasmic gene does not contain the sterile gene, and the nuclear gene does not contain the fertility restoring gene, which is fertile and can be self-bred. When the male sterile line plants were crossed with the maintainer line plants, the genetic composition of the offspring was the same as that of the male sterile line (S/rr), and it was still male sterile. The restorer plant nuclear gene contains fertility restoring gene (RR), and the cytoplasmic gene has or without sterile gene, which is fertile. The offspring of crossing with male sterile line (male sterile line) have genetic diversity (S/Rr) of male sterile line and restorer line, and have heterosis (Fig. 2). The cytoplasmic male sterile gene shows typical maternal inheritance, but the fertility-restoring gene in the nuclear genome can restore the male sterility caused by the cytoplasmic male sterile gene. According to the mechanism of CMS male sterility and the relationship between restoration and maintenance, rice CMS can be divided into three types: wild abortive type (WA), Baodai type (BT), and Honglian (HL).

### 3.1 Wild abortive cytoplasmic male sterility

In November 1970, Yuan Longping's team found a wild rice plant with pollen abortion in Sanya, which was later named “Wild abortive”. As a result, a wild abortive cytoplasmic male sterile line (WA-CMS) was successfully bred, which marked a breakthrough in rice heterosis research in China. The cytoplasm of WA-CMS comes from wild rice and belongs to sporophytic sterility. Most of the early indica rice varieties in the Yangtze River Basin were crossed and backcrossed with WA-CMS male sterile lines, and their progenies were sterile, while Indonesia Paddy Valley and Southeast Asian varieties Pitai had the ability to restore WA-CMS [].

WA-CMS is controlled by a mitochondrial chimeric gene encoding 352 amino acids, which is named WA352. WA352 is constitutively expressed in rice tissues. However, the encoded WA352 protein was only accumulated in tapetum cells at the pollen mother cell stage. WA352 protein can interact with nuclear coding protein COX11, and COX11 protein has the function of scavenging intracellular reactive oxygen species. The interaction between WA352 and COX11 inhibits the ability of COX11 to scavenge reactive oxygen species, resulting in the damage of reactive oxygen species in tapetum cells, leading to programmed cell death in advance in the pollen mother cell stage, unable to carry out normal meiosis, resulting in pollen abortion, which is characterized by classic failure [[12], [13], [14]].

WA-CMS has two fertility restorer genes: *Rf3* and *Rf4*. *Rf3* is located on rice chromosome 1 and has not been cloned. It is located between SSR markers RM443 and RM315, and the genetic distances between them are 4.4 and 20.7 cm, respectively. Previously reported that there was no WA352 accumulation in the restorer line ZSR1, but the content of WA352 transcripts was normal [14]. It is speculated that *Rf3* functions at the WA352 translation or post-translation level [14]. *Rf4* is located on rice chromosome 10 and has been successfully cloned. *Rf4* encodes a PPR protein that can degrade the WA352 transcript and reduce its abundance to about 20% of the original, thus restoring the fertility of WA-CMS [15,16].

### 3.2 Baotai type cytoplasmic male sterility

In 1966, Japanese scholar Shinjyo used Indian indica rice (Chinsurah Boro Ⅱ) as a female parent to cross and backcross with Taizhong 65, a japonica rice from Chinese Taipei, and selected a male sterile line Taichung 65 A from its progenies, from which a Baotai type cytoplasmic male sterile line (BT-CMS) was bred. BT-CMS cytoplasmic male sterile gene originated from Indian indica rice and belongs to gametophytic male sterility. Pollen abortion occurs in the late binucleate stage or trinucleate stage, and iodine staining is blue-black, which is a type of abortive male sterility [17]. Most japonica rice varieties can be used as maintainers of BT-CMS male sterile lines, while Southeast Asian indica rice and high-altitude indica rice varieties can be used as restorer lines of BT-CMS male sterile lines [18].

The BT-CMS sterile gene, orf79, is located downstream of the mitochondrial gene atp6 and is co-transcribed with the abnormal copy of atp6. The translated orf79 protein is cytotoxic and accumulates specifically in the microspores of the late binucleate or trinucleate stage, resulting in the microspore abortion of BT-CMS [19]. *RF1A* and *RF1B* are BT-CMS fertility restorer genes, which are members of a polygene cluster located at Rf-1 locus on rice chromosome 10, encoding PPR proteins with 18 and 11 PP R conserved domains, respectively. Among them, *RF1A* could directly cut the orf79 transcript of 2.0 kb into 1.5 and 0.5 kb fragments, and *RF1B* could completely degrade the orf79 transcript in the absence of *RF1A* and restore the fertility of BT-CMS. *RF1A* and *RF1B* silence orf79 through two different mechanisms, and *RF1A* has an epistatic effect on *RF1B*.

### 3.3 Honglian cytoplasmic male sterility

Zhu Ying's bred HL-CMS with Hainan Hongmang wild rice as female parent and indica rice Liantangzao as male parent in 1974. HL-CMS cytoplasm is derived from Hainan Hongmang wild rice and belongs to gametophytic sterility. Pollen abortion occurs in the binucleate stage and belongs to around abortion. The restoration and maintenance relationship of HL-CMS is basically opposite to that of WA-CMS. The Southeast Asian variety Pitai has the ability to maintain HL-CMS, while the Progenies of early indica rice varieties crossing with HL-CMS in the Yangtze River valley are fertile [20].

The sterility gene of HL-CMS is highly homologous to orf79, because the homology with ORFH79 is as high as 97%, only the difference of 5 bases leads to the production of 5 different amino acids, and ORFH79 is also located in the downstream of atp6 [21]. But the difference is that there is a non-homologous sequence between 36 bp upstream of ORFH79 (interval with atp6), and ORFH79 has two modes of existence. One is co-transcribed with atp6 to form 2.0 kb long atp6-ORFH79, and the other is independent in the form of 0.5 kb long ORFH79 (s). At present, there are two main hypotheses about the mechanism of orf79 sterility. One is the hypothesis of toxic protein similar to orf79. The expression of ORFH79 protein in microorganisms will seriously affect the growth and reproduction of microorganisms, indicating that ORFH79 protein is a toxic protein [22]. Another hypothesis is the energy hypothesis. The interaction between ORFH79 protein and P61, a subunit of mitochondrial electron transport chain complex Ⅲ, was found by yeast two-hybrid, BiFC, and Co-IP methods, which decreased the biological activity of P61 and led to ROS burst and ATP energy decrease, resulting in pollen abortion [23]. In addition, other studies have shown that ORFH79 is constitutively expressed in roots, stems, leaves, flowers, seedlings, and calli, and has an important effect on the growth, salt tolerance, and drought tolerance of rice roots [24,25]. Therefore, the mechanism of HL-CMS sterility remains to be further studied.

Rf5 and Rf6 are HL-CMS fertility restorer genes, Rf5 and *RF1A* are homologous, but the fertility restoration mechanism of Rf5 is slightly different from that of *RF1A*. RF5 does not interact with atp6-ORFH79 transcripts, but interacts with a GPR162 rich in GPR protein to form RF5-GPR162 dimer, binds with atp6-ORFH79 transcripts, splices atp6-ORFH79 transcripts, thus restoring HL-CMS fertility [26]. Rf6 is located on rice chromosome 8 and has been successfully cloned. RF6 protein binds to OsHXK6 in mitochondria and promotes the completion of abnormally transcribed atp6-ORFH79, resulting in normal development of HL-CMS pollen [27].

Due to the nuclear-cytoplasmic interaction of cytoplasmic male sterility, only a small number of rice germplasm resources can be cultivated into male sterile lines, and the fertility of some male sterile lines is easy to fluctuate under high temperatures, which limits the free combination of three-line method to a certain extent. However, the discovery of cytoplasmic male sterility solved the problem of large-scale hybridization of self-pollinated rice for the first time, laid the foundation for the creation of three-line hybrid rice, and provided an effective way to make use of the heterosis of self-pollinated crops such as rice. It opened a breakthrough for the application and research of hybrid rice.

### 3.4 Other types of cytoplasmic male sterility

In addition to the three recognized types mentioned above, many kinds of cytoplasmic genic male sterility were reported, such as LD- CMS type, D1-CMS, Indonesian paddy valley type, D type, Java type, dwarf abortive type, gang type, K type, and Ma Xie type.

In the 1960s, Japanese scholars discovered the cytoplasmic male sterile type LD-CMS determined by gametophyte from indica rice variety Lead Rice. The pollen sterility of LD- CMS type male sterile line was lower than that of BT- CMS type male sterile pollen, and the plant fertility was restored in sporophyte form by single nucleus coding gene Rf2 [28]. In LD-CMS type male sterile rice, mitochondrial gene orf79 plays a function as a cytoplasmic male sterile gene, which is similar to orf79 in rice derived from BT-CMS, but the content of orf79 protein is 1/20 of that in BT-CMS rice. The difference in orf79 protein level may be the main reason for the difference in pollen defect degree between LD-CMS and BT-CMS male sterile rice. The RF2 protein encoded by RF2 promotes the degradation of atp6-orf79 RNA in a form different from RF1. When Rf2 existed, the accumulation of orf79 in LD- CMS male sterile rice decreased to zero, thus the fertility was completely restored [29]. At present, the research on LD- CMS cytoplasmic sterility is mainly reported by Japanese scholars and focuses on the mechanism of sterility, while the breeding of corresponding male sterile lines and hybrid combinations is hardly reported in China.

In 1965, Professor Li Zhengyou of Yunnan Agricultural University selected from the hybrid progeny of japonica rice Taibei 8 and high altitude indica rice, D1-CMS determined by gametophyte, japonica rice red hat tassel sterile line [30]. Genetic analysis showed that the sterile genes and restorer genes of Dian I type male sterile line and BT-CMS male sterile line had an allelic effect and had the same mitochondrial chimeric gene atp6-orf79 [31,32]. Therefore, the pollen sterility control mechanism and fertility restoration mechanism of Dian I type male sterile line and BT-CMS male sterile line was the same. Up to now, Yunnan type I male sterile line is one of the main cytoplasmic male sterile lines for cultivating japonica hybrid rice combinations in China. According to the statistics of the National Rice data Center, there are 14 selected Yunnan type male sterile lines. In particular, Chunjiang series male sterile lines and Yongjing series male sterile lines are one of the most important genetic tools for heterosis utilization of indica stem crosses at present [[33], [34], [35], [36]].

D-type male sterile line is a cytoplasmic male sterile line determined by sporophyte, which is bred by the hybrid between DissiD52/37 and Aojiu Nante. The pollen abortion mode of this cytoplasmic male sterile type is the same as that of WA-CMS, and its sterility control gene is homologous [37]. D-type male sterile lines have high combining ability and good outcrossing habits. 21 male sterile lines have been approved or authorized, which plays a positive role in broadening the quality source of hybrid rice in China.

In addition, Java type, dwarf abortive type, gang type, K type and Ma Xie type all contain the same sterile gene as WA-CMS type [38], which belongs to sporophytic sterility. These six types of cytoplasmic male sterility also have a certain number of sterile lines approved or authorized (Table 1).

**Table 1:** Types and percentage of validated or authorized CMS lines over the years.

Types of CMS	Numbers of different types of CMS	Percentage (%)
WA-CMS	283	53.30%
BT-CMS	35	6.59%
HL-CMS	8	1.51%
D1 type	14	2.64%
Indonesian paddy valley type	52	9.79%
D type	21	3.95%
Java type	11	2.07%
Dwarf abortive type	11	2.07%
Gang type	12	2.26%
K type	4	0.75%
Ma Xie type	2	0.38%
Unknown	78	14.69%
Total	531	–

Note: The data come from the China Rice Data Center. The deadline is February 2020.

### 3.5 Application and limitation of cytoplasmic male sterile lines

At present, the sown area of the first-generation hybrid rice based on three-line method still accounts for more than half of the total sown area of hybrid rice, in which indica hybrid rice is mainly bred by WA- CMS type, Indian water type, D type and HL- CMS type, while BT-CMS male sterile line and Dian I type male sterile line are often used in japonica hybrid rice breeding due to the limitation of maintainer line background [[39], [40]]. In recent years, remarkable progress has been made in the study of the mechanism of cytoplasmic male sterility and its variety of breeding in rice. The discovery of many types of cytoplasmic male sterility has promoted its application in rice heterosis and hybrid production. However, the generation of cytoplasmic sterility depends on whether there is a fertility restorer gene in the nucleus under cytoplasmic sterile conditions, so it is impossible to breed all rice germplasm into cytoplasmic male sterile lines. According to the experience of breeders, only 0.1% of indica rice varieties can be transformed into cytoplasmic male sterile lines, and only 5% can be used as cytoplasmic male sterile lines, so the probability of using cytoplasmic male sterile lines to select good combinations is low. In addition, under the condition of high temperatures, the fertility of some cytoplasmic male sterile lines will be restored in a certain proportion. The above defects limit the further application of cytoplasmic male sterility in rice heterosis to some extent.

## 4 Environment sensitive nuclear male sterility

**Fig. 3:**
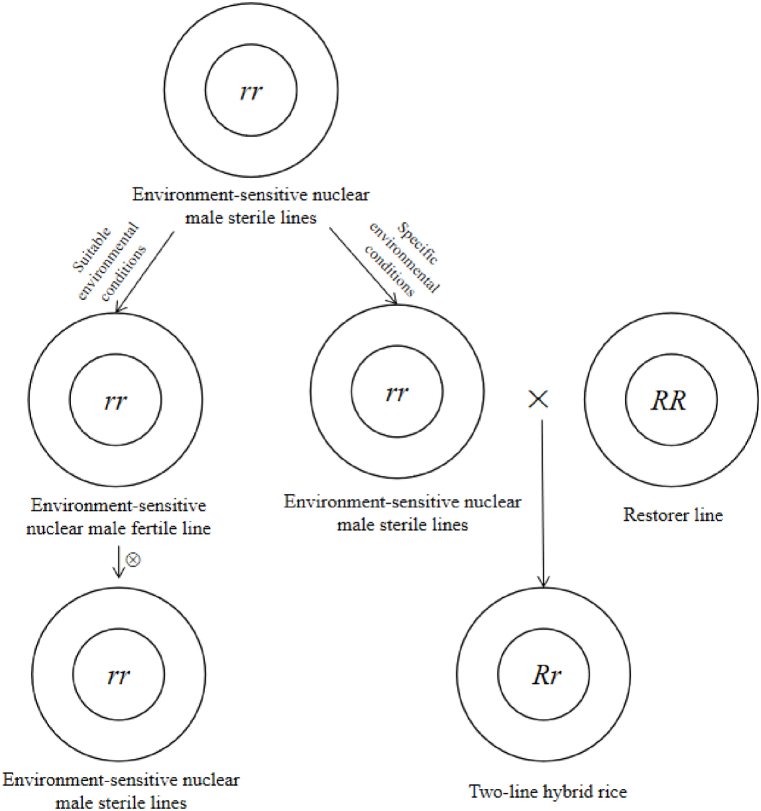
Schematic diagram of seed production system of two-line hybrid rice. r represents the environment-sensitive nuclear sterile gene, and R represents the nuclear fertility restoration gene.

Environment-sensitive nuclear male sterility (EGMS) is a kind of male sterility controlled by nuclear genes, and its fertility is easily affected by environment. Under suitable environmental conditions, EGMS is fertile, self-fertile and male sterile, while under specific environmental conditions, EGMS is male sterile and can be crossed with most conventional rice varieties (two-line restorer lines) to produce hybrids with heterosis (Fig. 3).

At present, it has been found that photoperiod, environmental temperature and environmental humidity have effects on the fertility of EGMS in rice. Therefore, according to the main factors affecting fertility transformation, EGMS can be divided into Photoperiod-sensitive genic male sterility (PGMS), Thermo-sensitive genic male sterility (TGMS) and Humidity-sensitive genic male sterility (HGMS). According to the main factors of the effect of photoperiod and temperature on the fertility transformation of male sterile lines, the photoperiod-sensitive and thermo-sensitive genic male sterile lines were divided into four types: long photoperiod sensitive genic male sterile type (photoperiod sensitive), short photoperiod sensitive genic male sterile type (reflective temperature sensitive), high-temperature genic male sterile type (temperature sensitive) and low-temperature genic male sterile type (inverse temperature sensitive). However, only low-humidity male sterility and high-humidity fertility have been found in humidity-sensitive genic male sterility. At present, 22 environmentally sensitive genic male sterile genes have been located in rice, of which 10 have been cloned and identified (Table 2).

**Table 2:** Types and genes of environmentally sensitive genic male sterility.

Gene		Chromosome	Sterile parents	Locus	Function	Reference
Long-photoperiod sensitive sterility	pms3	12	Nongken 58s	LOC_Os12g36030	Long-chain non-coding RNA	Mei et al. [41]
	pms1	7	Nongken 58s	KX578835	Long-chain non-coding RNA	Lu et al. [42]
	ptgms2-1	2	Guangzhan63 S	Unknown	Unknown	Xu et al. [43]
	pms2	3	32001S	Unknown	Unknown	Wang et al. [44]
	pms4	4	Mian9S	Unknown	Unknown	Huang et al. [45]
Short-photoperiod sensitive sterility	rpms1	8	Yi D1S	Unknown	Unknown	Peng et al. [46]
	rpms2	9	Yi D1S	Unknown	Unknown	Peng et al. [47]
	CSA	1	csa mutant	LOC_Os01g16810	MYB transcription factor	Zhang et al. [48]
High-temperature sensitive sterility	tms5	2	Annong S, Zhu 1 S	LOC_Os02g12290	RNase Z	Zhou et al. [49]
	p/tms12-1	12	Peiai 64 S	LOC_Os12g36030	small RNA	Zhou et al. [50]
	tms10	2	9522 S	LOC_Os02g18320	LRR-RLK	Yu et al. [51]
	Ugp1	9	Ugp1 co-inhibition plant	LOC_Os09g38030	UGP enzyme	Chen et al. [52]
	tms2	8	Norin-PL12	LOC_Os07g26940	Regulation of sphingolipid synthesis	Chutharat et al. [53]
	tms3	6	IR32364TGMS	Unknown	Unknown	Subudhi et al. [54]
	tms4	2	TGMS-VN1	Unknown	Unknown	Dong et al. [55]
	tms6	5	Sokcho-MS	Unknown	Unknown	Dong et al. [56]
	tms9-1	9	Hengnong S-1	Unknown	Unknown	Qi et al. [46]
Low-temperature sensitive sterility	tms6(t)	10	G20S	Unknown	Unknown	Jia et al. [57]
	rtms1	10	J207S	Unknown	Unknown	Liu et al. [58]
Humidity sensitive genic male sterility	OsOSC12	8	osc12 mutant	Os08g0223900	Triterpene synthase	Xue et al. [59]
	hms1	3	hms1 mutant	LOC_Os03g12030	Fatty acid synthase	Chen et al. [60]

### 4.1 Photoperiod sensitive genic male sterility

According to the fertility of rice under different photoperiod conditions, photoperiod sensitive genic male sterility (PGMS) can be divided into long photoperiod sensitive genic male sterile type and short photoperiod sensitive genic male sterile type. The long photoperiod sensitive genic male sterile type is male sterile in the long day environment and fertile in the short day environment, while the short photoperiod sensitive genic male sterile type is male sterile in the short day environment and fertile in the long day environment. At the same time, some studies have found that temperature also has a certain effect on PGMS, and high temperature will increase the sterility of PGMS [61,62].

At present, the long photoperiod sensitive genic male sterile genes identified are pms1, pms2, pms3 and pms4, which are mainly derived from japonica rice Nongken 58s and indica rice Guangzhan 63s. Previously reported that transferred Nongken 58s male sterile gene to indica photoperiod sensitive male sterile line 32001S by using RAPD and RELP markers, and identified two male sterile genes, pms1 located on chromosome 7 and pms2 on chromosome 3 [63]. The pms1 transcribes a long-stranded non-coding RNA (lncRNAs): PMS1T, PMS1T is preferentially expressed in young rice panicles and is recognized by miR2188 as a 21-nucleotide phasiRNA. Under long sunshine, phasiRNA accumulates in rice young panicles, leading to male sterility through some mechanism [64]. The pms2 gene has not been cloned and its sterility mechanism needs to be further studied. Previously reported that another photoperiod sensitive genic male sterile gene pms3, pms3 carried by Nongken 58s was located on rice chromosome 12 by BSA analysis [[65], [66]]. Then, it was found that pms3 also transcribed a lncRNAs:LDMAR, LDMAR under long-day irradiation, and there was only one SNP in the mutant and wild type, which may increase the methylation level of the promoter region of pms3 gene and change the secondary structure of LDMAR, resulting in the accumulation and decrease of LDMAR transcript specificity under long-day light, abnormal programmed anther death and long photosensitive sterility. Previously reported that crossed Mian 9s, a long photoperiod-sensitive male sterile line, with 6 indica rice varieties. Through map-based cloning, it was found that Mian 9s photoperiod-sensitive male sterile gene was located on chromosome 4 and named pms4 [45]. However, pms4 has not been cloned yet, and the related sterility mechanism needs to be further studied.

CSA, rpms1 and rpms2 have been identified as short photoperiod sensitive genic male sterile genes. In 2010, Zhang Bing's team found a small pollen carbon starved-mutant with white anthers. Through map-based cloning, the gene controlling this trait was located on chromosome 1, named Carbon starved anther (*CSA*), R2R3 MYB transcription factor [67]. Then it was found that *CSA* was highly expressed under short-day light and combined with rice monosaccharide transporter gene OsMST8 promoter to enhance the expression of OsMST8 and promote the unloading of photosynthate in anther. However, the sugar content of anthers decreased after the *CSA* gene mutation, resulting in abnormal pollen development and male sterility, while long light conditions promoted the high expression of the *CSA* homologous gene MYB5, leading to fertility restoration, so *CSA* mutants exhibited short photoperiod sensitive gene males sterile [48]. *Rpms1* and *rpms2* are derived from Yi D1S. At present, these two genes have not been cloned, and their sterility mechanism needs to be further studied [68]. Heat-sensitive gene male sterility is also an important part of environment-sensitive genic male sterility.

### 4.2 Thermo-sensitive genic male sterility

The fertility of thermosensitive genic male sterility (TGMS) is mainly regulated by environmental temperature, and the sensitive period of fertility transformation is from pollen mother cell formation to meiosis. According to the characteristics and performance of fertility transformation under different environmental temperatures, TGMS can be divided into high temperature-sensitive genic male sterility and low temperature-sensitive genic male sterility. The high temperature sensitive genic male sterile line is male sterile in the environment below the upper limit temperature of reproductive growth and above the critical temperature of fertility transformation, and fertile above the lower limit temperature of reproductive growth and below the critical temperature of fertility transformation. Low temperature-sensitive genic male sterility is opposite to high temperature-sensitive genic male sterility.

At present, two major genes of thermo-sensitive genic male sterility, tms5 and t/pms12-1 have been cloned. TMS5 encodes a conserved RNA enzyme ZS1, RNase ZS1 that can cleave and degrade three ubiquitin ribosomal L40 fusion protein gene UbL40 mRNA. In wild type, overexpression of UbL40 can lead to male sterility, and high temperature can increase the level of UbL40 mRNA in anther during the sensitive stage of pollen development. In the wild type, the excess UbL40 mRNA accumulated at high temperature could be cleaved by RNase ZS1 encoded by TMS5, and the anther development was normal. However, RNaseZS1 function is missing in tms5 mutants, and high temperature leads to excessive accumulation of UbL40 mRNA, which hinders the development of pollen and leads to pollen abortion [49,69]. The t/pms12-1 is allelic to pms3, but unlike pms3 regulating photoperiod-sensitive male sterility in japonica rice Nongken 58s (NK58S), t/pms12-1 regulates thermo-sensitive male sterility in indica rice Peiai 64s (PA64S). The t/pms12-1 mutant shows obvious thermo-sensitive genic male sterility. Wild-type T/PMS12-1 encodes a segment of non-coding RNA, resulting in a small 21 nt RNA, called osa-smR5864w. There is an SNP in mutant t/pms12-1, and the small RNA produced is osa-smR5864 m. The small RNA is preferentially expressed in young panicles and is not significantly affected by photoperiod and temperature. Overexpression of the non-coding RNA in PA64S could produce osa-smR5864w and restore pollen fertility of Peiai 64s, indicating that wild-type osa-smR5864w could regulate the expression of downstream pollen development genes, while mutant osa-smR5864 m lost its regulatory function, resulting in thermo-sensitive sterility [70]. At present, there are few reports about low temperature-sensitive genic male sterility, only in J207S and G20S, which is controlled by recessive genes rtms1 and tms6, respectively. But rtms1 and tms6 (t) has not been cloned, and the mechanism of male sterility needs to be further studied [57,58]. Humidity-sensitive gene male sterility also plays an important role.

### 4.3 Humidity sensitive genic male sterility

Humidity sensitivity genic male sterility (HGMS) is male sterile in low humidity environment and fertile in high humidity environment. As early as 2003, it was reported that humidity-sensitive male sterility was found in *Arabidopsis thaliana*. The pollen surface of the mutant was smooth and there was no obvious grid. Further studies found that the smooth appearance was due to tyrosine filling the outer cavity and covering the pollen surface, and the lack of sporopollen in the pollen exine, resulting in pollen inactivation of the mutant under low humidity conditions [71]. Humidity-sensitive male sterile lines have also been found in rice in recent years, and related genes such as *OsCER1* (*OsGL1-4*), *OsOSC12* and *OsHMS1* have been reported.

Rice *OsCER1* gene is the homologue of Arabidopsis wax synthesis gene CER1. *OsCER1* is specifically expressed in the tapetum of rice anther development S10–S11. In *OsCER1* antisense RNA lines, programmed death of tapetum was delayed, resulting in pollen abortion and changes in the expression levels of genes and plastids related to lipid metabolism, indicating that *OsCER1* plays a key role in ultra-long chain lipid biosynthesis in rice, affecting plastid development and programmed cell death in rice tapetum [72]. Further study showed that the tyrosine content of pollen wall of *OsCER1* knockout line decreased significantly, which may be due to the low adhesion rate and germination rate of mutant pollen on stigma under low humidity condition, which showed male sterility, while under high humidity condition, the pollen adhesion rate and germination rate increased significantly, which was the reason for fertility [73].

*OsGL1-4* also has high homology with CER1. It is homologous to *OsCER1* and is preferentially expressed in anthers and microspores of rice. *OsGL1-4* is located in endoplasmic reticulum. Mass spectrometric analysis showed that the contents of C25 and C27 alkanes in the anther exine of the mutant *OsGL1-4* were significantly lower than those of the wild type, which led to the vitality of the mutant pollen, but it was easy to dehydrate in the low humidity environment, resulting in very low pollen adhesion and germination rate, resulting in male sterility, while in the high humidity environment, the mutant pollen lost less water and the fertility was restored [[74], [75]].

*OsOSC12* encodes a conserved triterpene synthase in herbaceous plants. The “cereal tapetum alcohol” catalyzed by this enzyme is the main component of pollen coating. The pollen coating of mutant *OsOSC12* is defective, which results in the loss of protective layer function of pollen grains. Under the condition of humidity less than 60%, the pollen is rapidly dehydrated, resulting in male sterility, while it is completely fertile when the humidity is higher than 80%. And the rapid water loss of mutant pollen could be prevented by external application of linolenic acid and palmitic acid or stearic acid [59].

HMS1 encodes a β-ketoacyl-CoA synthetase, which plays a key role in the synthesis of very-long-chain fatty acids (VLCFAs) in rice. HMS1 catalyzes the biosynthesis of C26 and C28 VLCFAs, which contributes to the formation of rod-shaped structure and tyrosine in the pollen wall and protects the pollen from dehydration. Under the condition of low humidity, the adhesion of hms1 pollen to the stigma was poor and the germination decreased, while under the condition of high humidity, the character of pollen dehydration was supplemented and the fertility was restored. It was also found that the interaction between HMS1 and HMS1I, and the characters of mutant hms1i were basically consistent with those of mutant hms1 [76].

The survival of organisms needs a relatively stable environment. In the long process of evolution, organisms have produced many physiological mechanisms to adapt to a variety of external environmental changes. The environment-sensitive nuclear male sterility is caused by some related gene mutations that adapt to the changes of the external environment, which leads to the male sterility in some special environments. Making use of this characteristic that the fertility changes with the environment, the two-line method is not limited by the restoration-protection relationship, and it is simpler and freer than the three-line method, so it is an original way to utilize the heterosis of rice in China. However, when the environmental conditions fluctuate greatly, the fertility of environment-sensitive genic male sterile lines is easily affected, and there are certain risks in seed production, which limits the wide application of two-line method to a certain extent.

## 5 Male nuclear sterile rice and its mechanism of action

Rice stamen development and pollen formation are a series of complex physiological processes, which are regulated by many genes. At present, many common genic male sterile genes have been cloned, such as *OsMADS3* and *OsMADS16* regulating rice stamen meristem differentiation, *OsMSP1* and *MIL1* regulating the cooperation between microspore cells and anther wall cells [77,78]. *UDT1*, *TDR*, *EAT1*, *TIP2* and *PTC1* genes regulate tapetum development and programmed death of anther wall [[79], [80], [81], [82], [83], [84]], while *Wda1*, *CYP704B2* and *CYP703A3* genes regulate sporopollen and pollen wall formation [[85], [86], [87]]. The loss of function of related genes will lead to abnormal development of stamens or pollen, and can not be compensated by environmental factors. This kind of male sterility, which is controlled by recessive nuclear genes and is not affected by external environment, is called spontaneous genic male sterility (SGMS). The SGMS rice is not restricted by the relationship between restoration and protection, the combination is free, and the sterile character is stable, so it can be used in any environment where rice can grow normally, so it has a wider range of application and greater potential. However, due to the stable male sterility of common genic male sterile rice, there are difficulties in maintaining male sterile germplasm. Yuan Longping once proposed to use semi-maintainer lines to propagate common genic male sterile rice lines. However, this method can not effectively separate semi-maintainer lines from common genic male sterile lines [88]. In addition, some scholars have proposed to complete the reproduction of common genic male sterile lines by chemical male sterility, but the seed setting rate of chemical male sterility is low and the repeatability is poor, so the related techniques need to be further studied [89].

**Fig. 4:**
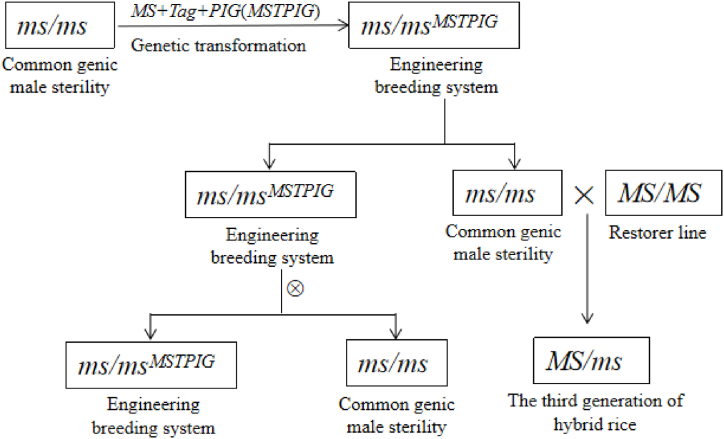
The process of engineering breeding system.

The more mature reproduction system of common genic male sterile lines is the engineering propagation system using genetic engineering technology at present. This method takes the common genic male sterile line as the background, genetically transforms a ternary linked expression vector of fertility restoring gene (MS), screening marker gene (Tag) and pollen inactivation gene (PIG), and obtains the engineering breeding line whose genetic group is ms/ms^MSTPIG^, in which the fertility restoring gene MS is responsible for compensating the sterile traits of the common genic male sterile line so that the engineering breeding line can produce fertile pollen. The pollen inactivation gene can just inactivate the pollen containing genetically modified components, and the engineering breeding line rice can only produce pollen without genetically modified components, namely ms pollen. The genetic group of inbred progeny of engineering breeding line is ms/ms or ms/ms^MSTPIG^, and the reproduction of common genic male sterile line and engineering breeding line is completed at the same time, and the ratio is 1: 1. The self-bred progenies of engineering breeding lines can be sorted quickly by screening marker gene Tag, and the progeny engineering breeding lines continue to complete the reproduction of common genic male sterile lines and engineering breeding lines through self-crossing. The third generation hybrid rice combinations with heterosis are produced by crossing common genic male sterile lines with restorer lines (Fig. 4).

Previously reported that transformed the recessive genic male sterile mutant ms26/ms26 (Wuyunjing 7) into a ternary linked expression vector composed of wild-type male sterile gene MS26, pollen inactivation gene ZM-aa1 and screening marker gene *DsRed2*, and realized the mass reproduction of common genic male sterile lines for the first time, and the sorting of male sterile line seeds and reproductive line seeds could be completed quickly by fluorescence sorting machine [90,91]. In 2016, Tang Xiaoyan and others obtained the recessive genic male sterile mutant osnp1/osnp1 by EMS mutagenesis, and then genetically transformed the linked wild-type genes OsNP1, Zm-aa1 and *DsRed2* to obtain the engineering breeding line Shenzhen 18 B. The common genic male sterile line Shenzhen 18 A passed the evaluation organized by the seed Management Station of Guangdong Province, China. The selected combinations Shenzhen 18 A/Yahui 2115 and Shenzhen 18 A/R278 completed regional trials in Sichuan and Guangdong, China, respectively [92]. Sanyou 1, the third generation hybrid rice bred by Li Xinqi team of Hunan Hybrid Rice Research Center in 2019, achieved a yield of 15 694.5 kg/hm2 per unit in Hengyang, Hunan Province, China. In 2020 and 2021, Sanyou 1 broke through the annual yield of 22 500 kg/hm2 of double-cropping late rice in the form of double-cropping late rice, which achieved good results in production and application demonstration for the third generation hybrid rice [93,94]. In 2021, Li's team at Hunan Hybrid Rice Research Center in China knocked out *CYP703A3* of 9311 by CRISPR-CSA9 technology, created a male sterile line 931103a3, constructed a pollen inactivation system using cytoplasmic male sterile gene ORFH79, and further constructed a linked expression vector of *CYP703A3*, *RF1B-*ORFH*79* and *DsRed2*. After genetic transformation, engineering breeding line 9311-3 B and common genic male sterile line 9311-3 A were obtained, and several strong dominant combinations were selected on this basis [95]. This system innovates the use of cytoplasmic male sterile genes to construct pollen inactivation system, which provides more options for the construction of pollen inactivation system. Previously reported that the specific expression of glucose pyrophosphorylase gene in rice endosperm, which made the endosperm of engineering breeding lines containing transgenic components atrophy and lighter in weight. The seeds of common genic male sterile lines and engineering breeding lines can be separated by wind force to construct a new screening mechanism [96]. Compared with the fluorescence screening mechanism based on *DsRed2*, this screening mechanism has the advantages of higher efficiency and lower equipment cost.

The third generation hybrid rice is based on the common genic male sterile line and the genetic engineering technology is used to create the engineering breeding line, which solves the reproduction problem of the common genic male sterile line rice, and has the advantages of stable fertility and free combination. And the male sterile line materials propagated by engineering breeding lines do not contain transgenic components, which can avoid the current policy restrictions on transgenic genes. It is believed that with the popularization and application of related technologies in the future, the third generation of hybrid rice will add a new way to increase rice production in China.

## 6 Apomixis and new generation hybrid rice

With the continuous development of hybrid rice technology, Academician Yuan Longping proposed in 1987 that the utilization of rice heterosis should develop from complex to simple and efficiency from high to low. Finally, apomixis technology was used to achieve the strategic goal of one-line hybrid rice [97]. Apomixis refers to the asexual reproduction in which the female and male gametes of angiosperms do not have nuclear fusion, which is divided into vegetative reproduction and apomixis. Apomixis can be divided into three ways: haploid gamete apomixis, diploid gamete apomixis and adventitious embryo. Among them, the most significant for the utilization of rice heterosis is diploid gamete apomixis, so the apomixis often referred to by breeders refers to the apomixis of diploid gametes, that is, rice female gametes or male gametes do not undergo meiosis and develop directly from diploid gametes to seeds without fertilization [98].

The core technology of apomixis in rice is to establish a technical system for producing diploid female/male gametes and to induce diploid gametes to develop into dynamic seeds. After long-term research and technical accumulation, especially after the understanding of female gamete meiosis and the breakthrough of gene editing technology, Domestic and foreign scholars have recently made important progress in rice apomixis research. Previously reported that transformed the meiotic process of reproductive development into meiosis into mitosis (MiMe) by knocking out three rice meiosis control genes OsOSD1, PAIR1 and OsREC8, thus producing diploid male and female gametes consistent with parental cell genotypes [99]. The successful implementation of MiMe technology in rice paves the way for the first half of apomixis utilization in rice. In 2019, Sundaresan et al. discovered that BBM1 gene, a member of the AP2 family of transcription factors expressed in spermatocytes, could induce parthenogenesis by ectopic expression in oocytes. Subsequently, *S*-Apo plants were obtained by interrupting female gamete meiosis in BBM1 transgenic rice plants by MiMe technique. *S*-Apo plants could produce diploid female gametes, and diploid female gametes were successfully induced to develop into seeds by ectopic expression of BBM1 gene in order to complete the apomixis process. The percentage of apomixis seeds induced by *S*-Apo plants is about 26% and can be passed on to offspring [100]. At the same time, on the basis of MiMe technology, Wang Kejian's team at China Rice Research Institute constructed rice Fix (Fixation of hybrids) by knocking out *OsMATL* gene and inducing the degradation of male gamete genomes. In the process of self-crossing of Fix plants to form progenies, diploid female gametes and male gametes were prevented by meiosis. During fertilization, the diploid male gamete has a 6% chance of genomic degradation to produce a zygotic embryo containing only the female gamete genome, because the diploid female gamete genome is consistent with the F1 parent in genotype. Therefore, the genome of the zygotic embryo is also consistent with the genotype of the F1 parent, thus realizing the cloning of the F1 hybrid embryo [101,102].

**Fig. 5:**
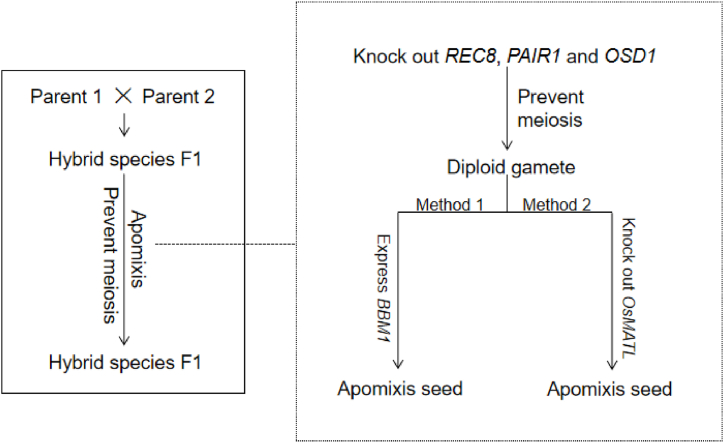
One-line system and F1 seeds apomixes.

Based on MiMe technology, two apomixis strategies of ectopic expression of *BBM1* gene and knockout of *OsMATL* gene show breeders the dawn of fixed heterosis in rice (Fig. 5). From the induction rate of non-fusion seeds, ectopic expression of BBM1 gene strategy is better than knockout *OsMATL* gene strategy, but the seeds propagated by ectopic expression BBM1 gene strategy contain transgenic components, so from the direction of industrial development in the future, knockout *OsMATL* gene strategy may have more potential for popularization and application. However, under the current technical conditions, the probability of apomixis seeds induced by the above two strategies is too low, and most of the seed embryos are tetraploid. Therefore, the one-line hybrid rice based on apomixis is still far away from practical application [103].

## 7 Discussion

The discovery of male sterility in rice has successfully promoted the development of heterosis utilization in rice and made a great contribution to global food security and economic construction. At present, the sown area of three-line hybrid rice based on cytoplasmic male sterility accounts for more than half of the sown area of hybrid rice. However, the backbone male sterile lines and restorer lines of Chinese three-line hybrid rice mainly belong to two heterosis groups, such as South Asia, Southeast Asia medium indica ecotype and South China early indica ecotype, and the recovery spectrum is narrow [104]. Recently reported that FA182 is a new cytoplasmic source which is different from the existing restoration and maintenance relationship, and has the characteristics of complete sporophytic sterility and single fertility restoration gene [105,106]. Strengthening the research on the application of FA182/OsRf19 system in breeding is of great significance to broaden the restoration spectrum and solve the limitations of three-line hybrid rice.

Compared with the three-line method, the two-line method has a wide range of restoration sources, is not restricted by the restoration-protection relationship, and the allocation of groups is freer. It has promoted the development of super high yield, high quality, green and light simplification of rice production in China. However, the fertility of two-line male sterile lines is easily affected by environmental factors such as light and temperature, and the sterility threshold temperature may gradually increase with the increase of male sterile line breeding generations, which makes the two-line male sterile line Gradually lose their usefulness. This poses a severe challenge to the development of two-line hybrid rice. Yuan Longping once proposed that “core seed production method” played a certain role in controlling the drift of sterile threshold temperature, but this method did not fundamentally solve the dilemma of increasing sterile threshold temperature generation by generation [107]. The genetic basis of sterile threshold temperature traits is not pure or genetic heterozygosity is the internal cause of genetic drift [108]. However, at present, there is no related genes reported, and the related research progress is slow. Therefore, on the one hand, the combination of anther culture and low-temperature selection can be adopted to select two-line male sterile lines with stable sterility expression, and on the other hand, the study on the genetic model of sterility threshold temperature can be strengthened. It is of great significance to guide the breeding of low sterile starting temperature male sterile lines with stable fertility.

The engineering breeding lines of the third generation hybrid rice can be quickly created by genetic engineering technology, which solves the reproduction problem of common genic male sterile lines, and the common genic male sterile lines have the advantages of stable fertility of three-line male sterile lines and free combination of two-line male sterile lines. Although the common genic male sterile lines used in seed production do not contain genetically modified components, the third generation hybrid rice involves genetic engineering technology, and there is still a lot of work to be done in national policy and public acceptance. Therefore, popular science propaganda should be increased to dispel people's doubts and misunderstandings, so as to promote national policy support for the development of the third generation hybrid rice.

In the article “Hybrid Rice Development Strategy” in 2018, Yuan Longping proposed five strategic development stages of hybrid rice: three-line method, “double-line method”, third-generation hybrid rice, carbon 4 (C4) hybrid rice, And the single-row method of hybrid rice. C4 type hybrid rice is to transform C3 type rice into C4 type in order to improve the photosynthetic capacity of rice and then greatly increase the yield of rice. The one-line method is the highest stage of the development of hybrid rice, which can produce hybrids whose characters never separate through apomixis technology, so as to fix the heterosis of rice. At present, Chinese hybrid rice has experienced a rapid development from three-line method to two-line method, and is actively promoting to the third generation of hybrid rice. Although C4 hybrid rice and one-line hybrid rice are still under research and exploration, it is believed that with the unremitting efforts of rice researchers and the continuous progress of molecular technology, this goal will eventually be achieved.

## Author contribution statement

Yusheng Xu and Dong Yu: Analyzed and interpreted the data, Performed the experiments and Wrote the paper.

Dong Yu and Jin Chen: Conceived and designed the experiments, Performed the experiments and Analyzed and interpreted the data.

Meijuan Duan: Contributed reagents, materials, analysis tools or data and Analyzed and interpreted the data.

## Data availability statement

Data included in article/supplementary material/referenced in article.

## Additional information

No additional information is available for this paper.

## Declaration of competing interest

The authors declare that they have no known competing financial interests or personal relationships that could have appeared to influence the work reported in this paper.
